# Gene Variation of Endoplasmic Reticulum Aminopeptidases 1 and 2, and Risk of Blood Pressure Progression and Incident Hypertension among 17,255 Initially Healthy Women

**DOI:** 10.1155/2018/2308585

**Published:** 2018-04-19

**Authors:** Robert Y. L. Zee, Alicia Rivera, Yaritza Inostroza, Paul M. Ridker, Daniel I. Chasman, Jose R. Romero

**Affiliations:** ^1^Division of Preventive Medicine, Brigham and Women's Hospital and Harvard Medical School, Boston, MA 02115, USA; ^2^Department of Pediatric Dentistry, Tufts University School of Dental Medicine, Boston, MA 02111, USA; ^3^Division of Endocrinology, Diabetes, and Hypertension, Department of Medicine, Brigham and Women's Hospital and Harvard Medical School, Boston, MA 02115, USA; ^4^Division of Nephrology, Vascular Biology Research Center, Department of Medicine, Beth Israel Deaconess Medical Center and Harvard Medical School, Boston, MA 02111, USA

## Abstract

Recent studies have demonstrated the importance of endoplasmic reticulum aminopeptidase (ERAP) in blood pressure (BP) homeostasis. To date, no large prospective, genetic–epidemiological data are available on genetic variation within ERAP and hypertension risk. The association of 45 genetic variants of *ERAP1* and *ERAP2* was investigated in 17,255 Caucasian female participants from the Women's Genome Health Study. All subjects were free of hypertension at baseline. During an 18-year follow-up period, 10,216 incident hypertensive cases were identified. Multivariable linear, logistic, and Cox regression analyses were performed to assess the relationship of genotypes with baseline BP levels, BP progression at 48 months, and incident hypertension assuming an additive genetic model. Linear regression analyses showed associations of four tSNPs (*ERAP1*: rs27524; *ERAP2*: rs3733904, rs4869315, and rs2549782; all *p* < 0.05) with baseline systolic BP levels. Three tSNPs (*ERAP1*: rs27851, rs27429, and rs34736, all *p* < 0.05) were associated with baseline diastolic BP levels. Multivariable logistic regression analysis showed that *ERAP1* rs27772 was associated with BP progression at 48 months (*p* = 0.0366). Multivariable Cox regression analysis showed an association of three tSNPs (*ERAP1*: rs469783 and rs10050860; *ERAP2*: rs2927615; all *p* < 0.05) with risk of incident hypertension. Analyses of dbGaP for genotype–phenotype association and GTEx Portal for gene expression quantitative trait loci revealed five tSNPs with differential association of BP and nine tSNPs with lower ERAP1 and ERAP2 mRNA expression levels, respectively. The present study suggests that ERAP1 and ERAP2 gene variation may be useful for risk assessment of BP progression and the development of hypertension.

## 1. Introduction

Elevated blood pressure is an important risk factor for the development of stroke, heart failure, and cardiovascular and renal disease. However, elevated blood pressure is controlled in only 54% of the US population with hypertension [1]. This is due, in part, to the fact that the pathophysiology of elevated blood pressure/hypertension is not entirely clear.

Endoplasmic reticulum aminopeptidase-1 (ERAP1; gene ID 51752, Chr. 5q15) and -2 (ERAP2; gene ID 64167, Chr. 5q15) are multifunctional aminopeptidases that have been proposed to play important roles in the pathophysiology of inflammatory and immune disorders associated with the major histocompatibility complex class I (MHC-I), preeclampsia, and hypertension [2–4]. ERAP1 and ERAP2 are zinc metallopeptidases that are members of the M1 oxytocinase subfamily that work in concert to catalyze the cleavage of amino acids from the N-terminus of various human antigens and peptide hormones [4–6] and have been shown to be widely expressed in human tissue including the heart, endothelial cells, and kidney. They are proposed to regulate blood pressure by inactivation of angiotensin II (AngII).

*In vitro*, ERAP1 catalyzes the conversion of AngII to angiotensin III and IV [5], and ERAP2 converts angiotensin III to angiotensin IV [6]. Recent studies have shown that ERAP1 binds to thioredoxin ERp44 within the endoplasmic reticulum and that global ablation of ERp44 in mice leads to increased circulating levels of ERAP1 and reduced blood pressure and AngII levels *in vivo*—results that are consistent with a role of ERAP1 in blood pressure homeostasis [7]. Furthermore, genetic–molecular approaches have identified variants among *ERAP1* and *ERAP2* genes that are associated with preeclampsia [8, 9], hemolytic uremia [10], and hypertension [11]. In particular, an association between the *ERAP1* rs30187 gene variant and hypertension was reported in a small cohort of 143 hypertensive and 348 normotensive Japanese subjects. Of importance, this variant was associated with reduced trimming efficiency of ERAP1 to inactivate AngII and increase bradykinin levels [12]. Moreover, there is evidence showing that hypertensive carriers of this genetic variant with left ventricular hypertrophy have significantly better left ventricular mass index responses to the AngII type 1 receptor antagonist than noncarriers have—results that implicate AngII and ERAP1 as potential regulators of left ventricular mass [13]. However, case–control genetic association analyses of the MRC British Genetics of Hypertension study participants showed *no* association between genetic variants at the *ERAP1* locus and essential hypertension in 1700 hypertensive and 1700 normotensive subjects [14, 15]. Furthermore, genetic variants of ERAP loci have not been reported to be associated with blood pressure in genome-wide association studies [16–20].

However, to date, no systematic, prospective epidemiological data are available that examine the relevance of the *ERAP1* and *ERAP2* gene loci as risk markers for hypertension. We thus evaluated the potential association of 33 *ERAP1* and 12 *ERAP2* tagging single-nucleotide polymorphisms (tSNPs) with (i) baseline systolic and diastolic blood pressure, (ii) blood pressure progression, and (iii) incident hypertension in a large prospective cohort of 17,255 initially healthy US white women.

## 2. Materials and Methods

### 2.1. Study Design

Details of the study design have been previously described [21, 22]. In brief, participants in the Women's Genome Health Study (WGHS)—a genetic substudy of the Women's Health Study [23, 24]—included initially healthy North American women aged 45 or older with no previous history of cardiovascular disease, cancer, or other major chronic illnesses. A baseline blood sample was collected during the enrollment phase of the Women's Health Study between 1992 and 1995. Study participants, who gave an informed consent for blood-based analyses related to risks of incident chronic diseases, were followed up for incident events that were adjudicated by an endpoints committee using standardized criteria and a full medical record review [23, 24]. The present investigation included 17,255 Caucasian participants of the WGHS; all were free of known cardiovascular disease, cancer, and hypertension at baseline. During a median follow-up time of 11.46 years (interquartile range: 6.52 to 18.68 years) for this sample population, a total of 10,216 newly diagnosed hypertensive cases were identified. The Brigham and Women's Hospital Institutional Review Board for Human Subjects Research approved the study protocol.

### 2.2. Study Variables

Blood pressure at randomization was self-reported by the participants, a group where self-report of blood pressure has proven highly accurate [25–27]. Women were classified into 3 predefined blood pressure categories: <120 mmHg for systolic and 75 mmHg for diastolic blood pressure; 120 to 129 mmHg for systolic or 75 to 84 mmHg for diastolic blood pressure; and 130 to 139 mmHg for systolic or 85 to 89 mmHg for diastolic blood pressure [28]. Women with discordant systolic and diastolic blood pressure categories were classified into the higher category. Covariables of interest were ascertained at study entry and included age, smoking status, history of hyperlipidemia (≥240 mg/dL or 6.22 mmol/L), body mass index (BMI; weight in kilograms divided by the square of height in meters), history of diabetes, frequency of exercise, alcohol consumption, and highest education level achieved.

### 2.3. Outcome Assessment

To assess blood pressure progression, we created categories of self-reported blood pressure at 48 months of follow-up identical to those at baseline as previously described [21, 22]. Blood pressure progression was defined by progressing ≥1 blood pressure category compared with baseline or by a new diagnosis of hypertension during the first 48 months. Incident cases of hypertension were defined by meeting ≥1 of the following criteria: self-report of a new physician diagnosis of hypertension assessed at years 1 and 3 and yearly thereafter; self-report of antihypertensive treatment assessed at years 1, 3, and 4; or self-reported systolic blood pressure of ≥140 mmHg or diastolic blood pressure of ≥90 mmHg assessed at years 1 and 4. Women reporting a new physician diagnosis of hypertension also provided month and year of diagnosis. For a diagnosis defined by another criterion or a missing date for a physician diagnosis, a date between the current and the previous questionnaire was randomly assigned. Women who developed cardiovascular disease, for which the management may affect blood pressure levels, were censored at the date of diagnosis and not considered at risk for incident hypertension thereafter.

### 2.4. Genotype Determination

As described elsewhere, DNA extracted from the baseline WGHS blood samples underwent tSNP (*r* ^2^ ≈ 0.80) genotyping using the genome-wide Illumina Infinium II HumanHap300 panel [29, 30]. Genotyping call rates were >99% per SNP.

### 2.5. GTEx mRNA Expression Profile in Cardiovascular Tissues

To determine the relationship between gene variants and mRNA expression levels, we explored publicly available Expression Quantitative Trait Loci (eQTL) data [31] for *ERAP1* and *ERAP2* in human coronary and tibial arteries, adrenal gland, and left ventricle tissues. The eQTL data presented were obtained from the Genotype-Tissue Expression (GTEx) Project consortium: GTEx Analysis Release V6p (dbGaP Accession phs000424.v6.p1). Effect estimates and *p* values were directly extracted from the GTEx dataset summary statistics report (https://gtexportal.org/home/).

### 2.6. Quantitative Real-Time PCR (qPCR) in Human Endothelial Cells

No (*in vitro*) data are available on the interplay between various blood pressure regulatory peptides and ERAPs in relation to expression levels. *In vitro*, AngII activates endothelial cells, leading to increased angiogenesis and reactive oxygen species production among other effects [32–34]. For the present *in vitro* studies, the human endothelial cell line, EA.hy926 (American Type Culture Collection: CRL-2922), was used; this cell line was previously documented by us and others to be responsive to AngII and aldosterone activation [32–34]. The effects of AngII on ERAP1 as well as ERAP2 gene expression were investigated. In brief, cells were grown in 10% fetal bovine serum-Dulbecco's Modified Eagle Medium and split 1 : 16 at confluence [33, 34]. Cells were then treated for 24 hours with AngII (10 nM) in the presence or absence of losartan (1 *μ*M), an AngII type I receptor antagonist. Total RNA was extracted using the RNeasy Mini kit (Qiagen Sciences, Hilden, Germany) following the manufacturer's instructions. cDNA was synthesized from 3 *μ*g of total RNA with the First Strand cDNA Synthesis kit (Amersham, Little Chalfont, United Kingdom). PCR amplification reactions were performed with TaqMan gene expression assays for ERAP1 (Hs00429970_m1) and ERAP2 (Hs01073631_m1) in triplicate with the ABI Prism 7000 Sequence Detection System (Applied Biosystems, Foster City, CA). The cycle threshold method was used following the manufacturer's recommendation to determine mRNA levels. Target gene expression was normalized to glyceraldehyde 3-phosphate dehydrogenase (GAPDH) and 18S rRNA levels.

### 2.7. Statistical Analyses

Genotype frequencies were compared with values predicted by the Hardy–Weinberg equilibrium using the chi-square test with one degree of freedom. Multivariable linear regression analysis, adjusting for age, BMI, history of diabetes, history of hyperlipidemia, current smoking status, exercise, alcohol use, education level, and current (any) hormone use, was performed to assess the relationship of genotypes with baseline blood pressure measurements. Multivariable logistic regression analysis was performed to examine the associations between genotypes and blood pressure progression at 48 months, adjusting for age, BMI, history of diabetes, history of hyperlipidemia, current smoking status, exercise, alcohol use, education level, current (any) hormone use, and randomized treatment assignments. Hazard ratios (HRs) associated with each of the SNPs were calculated separately by Cox regression analysis, adjusting for age, BMI, history of diabetes, history of hyperlipidemia, current smoking status, exercise, alcohol use, education level, current (any) hormone use, and randomized treatment assignments. The proportional hazards assumption was examined for all of the models by including a genotype by logarithm of time interaction into each model. All analyses were carried out using SAS v9.1 package (SAS Institute Inc.) or R software, assuming an additive model for genetic effects. Because of the confirmatory nature of the current study and the extended data from several public consortia, a 2-tailed uncorrected (for multiple testing) *p* value of 0.05 was considered a statistically significant result.

## 3. Results

### 3.1. ERAP1 and ERAP2 Variants with Baseline Blood Pressure, Blood Pressure Progression, and Risk of Hypertension

The baseline characteristics of the 17,255 initially healthy Caucasian women are shown in Table 1. Two (rs17482078 and rs25866) out of the 45 SNPs evaluated were not in the Hardy–Weinberg equilibrium with uncorrected *p* values <0.001. Results from the linear regression analyses showed evidence for differential associations of four SNPs (*ERAP1*: rs27524; *ERAP2*: rs3733904, rs4869315, and rs2549782; all *p*-uncorrected <0.05) with baseline systolic blood pressure levels (Online Supplementary Data Table 1) and three SNPs (*ERAP1*: rs27851, rs27429, and rs34736, all *p*-uncorrected <0.05) with baseline diastolic blood pressure levels (Online Supplementary Data Table 2), respectively. In the multivariable logistic regression analysis, *ERAP1* rs27772 was shown to be associated with blood pressure progression at 48 months (*p*-uncorrected = 0.0366; Table 2). Results from the multivariable Cox regression analysis showed evidence for an association of three SNPs (*ERAP1*: rs469783 and rs10050860; *ERAP2*: rs2927615; all *p*-uncorrected <0.05) with risk of incident hypertension (Table 3). All SNPs evaluated were in agreement of proportionality hazard assumption.

### 3.2. mRNA Expression Profile in Cardiovascular Tissues by ERAP Gene Variants

A total of 12 *ERAP* SNPs that showed significant effects in the present study were evaluated (for *ERAP1*: rs27524, rs27851, rs27429, rs30187, rs34736, rs27772, rs469783, and rs10050860; for *ERAP2*: rs3733904, rs4869315, rs2549782, and rs2927615) [31]. In the tibial artery, all *ERAP1* genetic variants were associated with significantly decreased *ERAP1* mRNA expression (*p* < 0.0025) except for rs27851 that showed an increase (Supplementary Data Table 3). In all other tissues analyzed, at least four of the SNPs were associated with decreased *ERAP1* mRNA levels. With regards to *ERAP2*, with the exception of rs2927615, all other variants were significantly associated with reduced *ERAP2* mRNA in the four tissue types tested.  Table 4 sequentially presents the association of the GTEx SNP list with the phenotypic outcomes examined in the present study. The overall findings suggest that genetic variants of *ERAP1* and *ERAP2* that were associated with blood pressure homeostasis may be predictive of lower *ERAP1* and *ERAP2* expression levels.

We examined ERAP1 and ERAP2 gene expression levels in the human endothelial cell line, EA.hy926, that were stimulated with AngII. We observed that AngII incubation significantly increased *ERAP1* but not *ERAP2* mRNA levels (Supplementary Data Figure 1). The AngII-stimulated *ERAP1* expression was blocked by preincubation with losartan, an AngII type I receptor antagonist. Consequently, our present results suggest that endothelial cell activation is associated with the activation of the AngII type 1 receptor and increased expression of *ERAP1*.

## 4. Discussion

Angiotensin II is one of the principal effector molecules of the renin-angiotensin-aldosterone system (RAAS). RAAS plays a critical role in sodium, water homeostasis, and blood pressure regulation. Indeed, disordered RAAS activation is associated with endoplasmic reticulum stress, increased reactive oxygen species, and inflammation, thus contributing to the pathophysiology of stroke, hypertension, and heart failure [35–37]. Our results support the contention that *ERAP* loci play a role in blood pressure homeostasis and the development of hypertension. We provide evidence that the genetic variation of *ERAP* was associated with baseline blood pressure, blood pressure progression, and incident hypertension. In addition, *ERAP1* SNPs were associated with altered *ERAP1* mRNA levels in vascular and adrenal tissues [31]. We thus posit that disordered *ERAP1* levels may contribute to the pathophysiology of hypertension. Of importance, we report that *in vitro* activation of endothelial cells by AngII leads to increases in *ERAP1* mRNA via activation of the AngII type 1 receptor in endothelial cells (Supplementary Data Figure 1).

Differential associations of ERAP gene variants with various outcomes have been reported (Table 5), providing suggestive evidence for its involvement in blood pressure regulation. Taken together with our data, genetic variants of *ERAP*, in particular *ERAP1*, may modulate RAAS activity which in turn may regulate ERAP1 levels through a potential negative feedback mechanism. ERAP regulates bradykinin and AngII levels. However, further studies are needed to characterize this novel relationship between endoplasmic reticulum activation and ERAP1 and RAAS activation for its potential therapeutic applicability in blood pressure regulation.

As shown in Table 5, not all the published reports examined the same set of SNPs, nor did these studies comprehensively and simultaneously examine the association of ERAP gene loci with blood pressure progression, incident hypertension, and gene expression profile. Furthermore, not all published studies were conducted using comparable study design(s) or in similar racial/ethnic sample population(s), thus making a direct comparison and informative interpretation across studies difficult. Given this situation, a possible explanation for the apparent discrepancies is that the observed allele frequencies for the SNPs examined may differ between various studies, which could be due to population/ethnic differences/substructures.

Since several genome-wide association studies were conducted to determine the genetic risk factors for blood pressure [16–20], we further investigated the relationship using the Phenotype–Genotype Integrator in the NCBI dbGaP website [38–40]. Based on the dbGaP data repository, several SNPs within the ERAP1 and ERAP2 gene loci were reported to be associated with blood pressure (Supplementary Data Table 4), further indicating the potential involvement of ERAPs in blood pressure development.

The strengths of the present study are the overall sample size, the biological relevance of the polymorphisms considered, the prospective design, and the complete long-term follow-up among women. This is important as limited studies have addressed blood pressure outcomes in women exclusively and growing evidence shows that women are at a higher risk of developing hypertension-related cardiovascular diseases such as heart failure with preserved ejection fraction than men are [41]. We also chose, on an a priori basis, to present all our data simultaneously rather than focusing on any one specific finding. Nonetheless, potential limitations of our study require discussion. Limitations include generalizability and potential bias. We examined only Caucasian middle-aged and older women of distinct socioeconomic status (health professionals), and our findings may not be generalizable to other populations with diverse ethnicity or socioeconomic background. In our study, we had the ability to detect, based on the present sample sizes, assuming 80% power, at an alpha of 0.05, a hazards ratio of greater than 1.08 if the minor allele frequency is 0.50 and of greater than 1.09 if the minor allele frequency is 0.05 assuming a univariable-additive model. Thus, we cannot rule out a low risk associated with the SNPs tested. Finally, confirmation in other prospective studies is warranted. Nonetheless, our present findings and the collective data reported in the dbGaP consortium (Online Supplementary Data Table 4) provide confirmatory evidence for an association of ERAP1 and ERAP2 gene loci with blood pressure levels.

In conclusion, the present findings provide evidence for the involvement of ERAP1 and ERAP2 gene loci in blood pressure regulation and the pathogenesis of hypertension with an added indication of ERAP1 gene locus as a potential therapeutic target for blood pressure management.

## Figures and Tables

**Table 1:** Baseline characteristics of the study population.

Variable	*N* = 17,255
Age, years	52.00 [48.00, 57.00]
Body-mass index, kg/m^2^	24.21 [22.05, 27.40]
Smoking status, %	
Current	11.88
Past	37.24
Never	50.88
History of diabetes, %	1.29
History of hyperlipidemia ≥240 mg/dL, %	24.98
Exercise, times/week, %	
Rarely/never	35.19
<1	19.80
1 to 3	33.11
>3	11.89
Alcohol use, %	
Rarely/never	41.41
Monthly	13.45
Weekly	34.42
Daily	10.71
Highest education level, %	
Less than a Bachelor's degree	53.67
Bachelor's degree	24.97
Master's or doctoral degree	21.37
Aspirin use, %	49.71
Beta-carotene use, %	49.92
Vitamin-E use, %	50.38
Current hormone use, %	43.32
Baseline BP category, %	
<120/75 mmHg	44.76
120–129/75–84 mmHg	39.14
130–139/85–89 mmHg	16.10

Data are median and interquartile range for continuous, and percentages for categorical variables.

**Table 2:** Logistic regression analysis for all SNPs evaluated with blood pressure progression.

dbSNP	MA	MAF	OR	Lower 95% CI	Upper 95% CI	*p*-uncorrected
*ERAP1*						
rs1559085	G	0.1413	1.056	0.988	1.129	*0.1094*
rs27851	G	0.0607	0.946	0.858	1.043	*0.2686*
rs3756623	C	0.0750	0.970	0.888	1.059	*0.4924*
rs754615	C	0.3907	1.014	0.967	1.064	*0.5581*
rs1862609	A	0.3129	1.022	0.973	1.075	*0.3846*
rs27772	G	0.3133	0.948	0.901	0.997	*0.0366*
rs28081	A	0.1510	1.006	0.943	1.073	*0.8643*
rs27037	A	0.2957	1.040	0.988	1.094	*0.1314*
rs27429	C	0.0598	0.943	0.855	1.041	*0.2457*
rs27524	A	0.3730	1.036	0.987	1.087	*0.1497*
rs25862	A	0.4488	0.996	0.951	1.044	*0.8777*
rs11135480	C	0.1321	1.032	0.964	1.105	*0.3662*
rs10515247	A	0.1323	1.033	0.964	1.106	*0.3593*
rs149078	A	0.2965	0.975	0.927	1.026	*0.3280*
rs27042	A	0.3655	0.959	0.914	1.007	*0.0913*
rs27044	G	0.2795	1.025	0.973	1.079	*0.3557*
rs17482078	A	0.2039	1.039	0.981	1.101	*0.1883*
rs42398	G	0.1470	1.009	0.945	1.077	*0.7856*
rs469783	G	0.4374	1.016	0.970	1.065	*0.4956*
rs10050860	A	0.2127	1.026	0.969	1.086	*0.3862*
rs13154629	A	0.2131	1.035	0.978	1.096	*0.2276*
rs30187	A	0.3478	1.019	0.971	1.070	*0.4463*
rs27434	A	0.2175	1.003	0.948	1.062	*0.9069*
rs26618	G	0.2370	0.975	0.923	1.030	*0.3650*
rs25866	A	0.2341	0.998	0.943	1.055	*0.9369*
rs26653	C	0.2835	0.984	0.934	1.036	*0.5304*
rs34753	G	0.2796	0.979	0.930	1.031	*0.4259*
rs28129	G	0.2804	0.979	0.930	1.031	*0.4227*
rs18036	G	0.2441	0.982	0.930	1.037	*0.5188*
rs152280	A	0.2085	0.991	0.936	1.049	*0.7572*
rs12520537	G	0.1537	0.987	0.926	1.053	*0.6997*
rs41135	G	0.4669	1.016	0.970	1.064	*0.5092*
rs34736	A	0.0648	0.929	0.845	1.021	*0.1254*
*ERAP2*						
rs2911132	A	0.3726	1.014	0.967	1.064	*0.5638*
rs2042381	A	0.2733	1.022	0.970	1.077	*0.4111*
rs2927615	A	0.2404	1.027	0.973	1.084	*0.3339*
rs2927612	G	0.1168	0.993	0.924	1.067	*0.8550*
rs2549778	A	0.4292	1.005	0.959	1.053	*0.8417*
rs6861666	G	0.0781	1.014	0.930	1.106	*0.7579*
rs3733904	G	0.2117	0.965	0.911	1.022	*0.2239*
rs2549779	G	0.4880	0.983	0.939	1.030	*0.4843*
rs4869315	A	0.4241	0.969	0.924	1.015	*0.1846*
rs2549782	C	0.4093	1.047	0.999	1.097	*0.0569*
rs17408150	T	0.0560	1.050	0.948	1.162	*0.3470*
rs7714122	G	0.0648	0.999	0.908	1.098	*0.9794*

Adjusted for age, body mass index, history of diabetes, history of hyperlipidemia, current smoking, exercise, alcohol use, education level, current hormone use, and randomized treatment assignment. MA = minor; MAF = minor allele frequency; OR = odds ratio; CI = confidence interval.

**Table 3:** Cox regression analysis for all SNPs evaluated with incident hypertension.

dbSNP	MA	MAF	HR	Lower 95% CI	Upper 95% CI	*p*-uncorrected
*ERAP1*						
rs1559085	G	0.1413	0.997	0.957	1.037	*0.8652*
rs27851	G	0.0607	1.008	0.951	1.069	*0.7869*
rs3756623	C	0.0750	1.011	0.959	1.065	*0.6970*
rs754615	C	0.3907	0.996	0.968	1.025	*0.7774*
rs1862609	A	0.3129	0.990	0.961	1.021	*0.5240*
rs27772	G	0.3133	0.986	0.956	1.016	*0.3421*
rs28081	A	0.1510	1.011	0.973	1.051	*0.5739*
rs27037	A	0.2957	1.026	0.995	1.058	*0.0967*
rs27429	C	0.0598	1.008	0.95	1.070	*0.7860*
rs27524	A	0.3730	1.029	1.000	1.059	*0.0537*
rs25862	A	0.4488	0.992	0.965	1.021	*0.5849*
rs11135480	C	0.1321	1.021	0.980	1.064	*0.3230*
rs10515247	A	0.1323	1.021	0.980	1.064	*0.3261*
rs149078	A	0.2965	0.999	0.969	1.030	*0.9572*
rs27042	A	0.3655	0.996	0.968	1.026	*0.8056*
rs27044	G	0.2795	1.013	0.982	1.045	*0.4108*
rs17482078	A	0.2039	0.980	0.947	1.015	*0.2539*
rs42398	G	0.1470	1.009	0.970	1.050	*0.6454*
rs469783	G	0.4374	1.032	1.003	1.062	*0.0291*
rs10050860	A	0.2127	0.964	0.931	0.997	*0.0351*
rs13154629	A	0.2131	0.973	0.940	1.007	*0.1182*
rs30187	A	0.3478	1.026	0.996	1.056	*0.0914*
rs27434	A	0.2175	1.026	0.992	1.062	*0.1403*
rs26618	G	0.2370	0.987	0.955	1.020	*0.4313*
rs25866	A	0.2341	1.015	0.982	1.050	*0.3732*
rs26653	C	0.2835	1.019	0.988	1.051	*0.2330*
rs34753	G	0.2796	1.016	0.985	1.048	*0.3224*
rs28129	G	0.2804	1.016	0.985	1.048	*0.3149*
rs18036	G	0.2441	1.000	0.968	1.033	*0.9944*
rs152280	A	0.2085	1.013	0.978	1.048	*0.4764*
rs12520537	G	0.1537	1.028	0.989	1.068	*0.1629*
rs41135	G	0.4669	0.981	0.953	1.009	*0.1740*
rs34736	A	0.0648	1.001	0.945	1.060	*0.9785*
*ERAP2*						
rs2911132	A	0.3726	0.986	0.958	1.015	*0.3506*
rs2042381	A	0.2733	1.010	0.979	1.042	*0.5209*
rs2927615	A	0.2404	0.958	0.927	0.990	*0.0107*
rs2927612	G	0.1168	0.991	0.949	1.036	*0.6977*
rs2549778	A	0.4292	0.998	0.970	1.026	*0.8664*
rs6861666	G	0.0781	1.028	0.976	1.082	*0.2971*
rs3733904	G	0.2117	0.991	0.957	1.026	*0.6043*
rs2549779	G	0.4880	0.994	0.966	1.022	*0.6693*
rs4869315	A	0.4241	0.987	0.960	1.016	*0.3795*
rs2549782	C	0.4093	1.019	0.991	1.048	*0.1873*
rs17408150	T	0.0560	1.046	0.985	1.111	*0.1450*
rs7714122	G	0.0648	1.030	0.974	1.090	*0.2986*

Adjusted for age, body mass index, history of diabetes, history of hyperlipidemia, current smoking, exercise, alcohol use, education level, current hormone use, and randomized treatment assignment. MA = minor; MAF = minor allele frequency; HR = hazard ratio; CI = confidence interval.

**Table 4:** Summary of association of the GTEx SNP list evaluated with the phenotypic outcomes examined in the present study.

*ERAP1*	Coronary^∗^ artery	Tibial^∗^ artery	Adrenal^∗^ gland	Left^∗^ ventricle	B-SBP^∗∗^	B-DBP^∗∗^	Prog^∗∗^	HTN^∗∗^
rs27524	0.0042	0.00087	<0.0001	<0.0001	0.0492	ns	ns	ns
rs27851	0.043	0.0023	0.13	0.66	ns	0.0083	ns	ns
rs27429	0.08	0.0025	0.53	0.55	ns	0.0093	ns	ns
rs30187	0.00022	<0.0001	<0.0001	<0.0001	ns	ns	ns	ns
rs34736	0.10	0.00016	0.23	0.58	ns	0.0175	ns	ns
rs27772	0.052	<0.0001	0.00028	0.18	ns	ns	0.0366	ns
rs469783	0.0042	<0.0001	0.002	0.00075	ns	ns	ns	0.0291
rs10050860	0.88	<0.0001	0.18	0.023	ns	ns	ns	0.0351
*ERAP2*								
rs3733904	<0.0001	<0.0001	0.0029	<0.0001	0.0090	ns	ns	ns
rs4869315	<0.0001	<0.0001	<0.0001	<0.0001	0.0108	ns	ns	ns
rs2549782	<0.0001	<0.0001	<0.0001	<0.0001	0.0320	ns	ns	ns
rs2927615	0.39	0.64	0.75	0.51	ns	ns	ns	0.0107

^∗^Values presented are *p* values for gene expression analysis from the GTEx Portal database (GTEx Analysis Release V6p). ^∗∗^Values presented are uncorrected *p* values reported in the present investigation. B-SBP = baseline systolic blood pressure; B-DBP = baseline diastolic blood pressure; Prog = blood pressure progression; HTN = incident hypertension; ns = *p* value >0.05.

**Table 5:** Cross-reference comparison for specific ERAP1 and ERAP2 gene variants.

	Present study	Yamamoto et al. [11]	Yang et al. [42]	Johnson et al. [9]	Johnson et al. [9]	Hill et al. [8]	Hill et al. [8] Preeclampsia Unpaired African-American
Outcome	Incident hypertension	Hypertension	Hypertension	Preeclampsia	Preeclampsia	Preeclampsia	Preeclampsia
Sample population	US White females	Japanese case/control	Northeastern Han Chinese case/control	Australian/New Zealand familial cohort	Norwegian case/control	Chilean maternal-fetal dyads	Unpaired African-American
Sample size	17,255	143/348	300/233	74 families	1139/2269	1103 maternal–fetal dyads	836 maternal 837 fetal
dbSNP							
*ERAP1*							
rs30187	HR = 1.026 95%CI = 0.996–1.056 *p* = 0.0914	OR = 1.6 95%CI = 1.2–2.3	—	—	—	—	—
rs26618	HR = 0.987 95%CI = 0.955–1.020 *p* = 0.4313	ns	—	—	—	—	—
rs27980	—	—	OR = 1.361 95%CI = 0.900–2.059 *p* = 0.144	—	—	—	—
rs17086651	—	—	OR = 1.660 95%CI = 1.025–2.686 *p* = 0.039	—	—	—	—
rs3734016	—	—	—	*p* = 0.009	—	—	
rs34750	—	—	—	—	*p* = 0.011	—	—
*ERAP2*							
rs2549782	HR = 1.019 95%CI = 0.991–1.048 *p* = 0.1873	—	—	*p* = 0.004	—	ns	OR = 1.320 95%CI = 1.075–1.619 *p* = 0.009
rs17408150	HR = 1.046 95%CI = 0.985–1.111 *p* = 0.1450	—	—	—	*p* = 0.009	ns	Not further evaluated due to allelic rarity

HR = hazard ratio; OR = odds ratio; CI = confidence interval; ns = nonsignificant.

## Additional Points

*Novelty and Significance*. What Is New? (i) Tagging single-nucleotide polymorphisms (tSNPs) of the endoplasmic reticulum aminopeptidase (ERAP) 1 and 2 is associated with baseline systolic and diastolic blood pressure (BP). (ii) Three ERAP1 and ERAP2 tSNPs are associated with risk of incident hypertension. (iii) dbGaP analyses showed a genotype–phenotype association with five tSNPs and BP. (iv) Gene expression quantitative trait loci analyses revealed that these tSNPs were associated with reduced ERAP1 and ERAP2 mRNA expression levels in human cardiovascular tissue. What Is Relevant? (i) ERAPs plays an important and well-described role in immune function as they trim HLA-binding precursors modulating their inclusion into MHC-I. (ii) ERAPs metabolize a variety of cell surface receptors and peptides including angiotensin-II. (iii) Small case–control studies provided evidence that polymorphic variants of ERAP1 and ERAP2 are associated with preeclampsia and hypertension. (iv) Recent data suggests that ERAP1 also is a mediator of the hypotensive response to sepsis. *Summary*. This large, prospective cohort study among initially healthy women supports the contention that ERAP gene variation may be useful for risk assessment of BP progression and the development of hypertension.

## Supplementary Materials

Supplementary Materials Online Supplementary Data Table 1: linear regression analysis for all SNPs evaluated with baseline systolic blood pressure levels. Online Supplementary Data Table 2: linear regression analysis for all SNPs evaluated with baseline diastolic blood pressure levels. Online Supplementary Data Table 3: expression quantitative trait loci (eQTL) of SNPs associated with blood pressure progression and incident hypertension—eQTL from mRNA expression of otherwise normal human cardiovascular tissues based on the ^∗^GTEx Analysis Release V6p (dbGaP accession phs000424.v6.p1). Online Supplementary Data Table 4: genotype–phenotype association of ERAP 1 and ERAP2 gene variants with blood pressure as described in dbGaP. Online Supplementary Data Figure 1: ERAP1 and ERAP2 gene expression levels in the human endothelial cell line, EA.hy926.
