# Supplementary material for: Gene Variation of Endoplasmic Reticulum Aminopeptidases 1 and 2, and Risk of Blood Pressure Progression and Incident Hypertension among 17,255 Initially Healthy Women
Source: Int J Genomics. 2018 Apr 19;2018:2308585. doi: 10.1155/2018/2308585 (PMC5933071; doi:10.1155/2018/2308585)
Supplement: Supplementary Materials — Online Supplementary Data Table 1: linear regression analysis for all SNPs evaluated with baseline systolic blood pressure levels. Online Supplementary Data Table 2: linear regression analysis for all SNPs evaluated with baseline diastolic blood pressure levels. Online Supplementary Data Table 3: expression quantitative trait loci (eQTL) of SNPs associated with blood pressure progression and incident hypertension—eQTL from mRNA expression of otherwise normal human cardiovascular tissues based on the ∗GTEx Analysis Release V6p (dbGaP accession phs000424.v6.p1). Online Supplementary Data Table 4: genotype–phenotype association of ERAP 1 and ERAP2 gene variants with blood pressure as described in dbGaP. Online Supplementary Data Figure 1: ERAP1 and ERAP2 gene expression levels in the human endothelial cell line, EA.hy926. [file 2308585.f1.pdf]

SUPPLEMENTAL MATERIAL

Online Supplementary Data Table 1: Linear regression analysis for all SNPs evaluated with baseline systolic blood pressure levels

dbSNP	MA	MAF	Beta-estimate	SE	t-Value	*p*-uncorrected
ERAP1						
rs1559085	G	0.1413	0.02971	0.13886	0.21	*0.8306*
rs27851	G	0.0607	0.31814	0.20335	1.56	*0.1177*
rs3756623	C	0.0750	0.07390	0.18380	0.40	*0.6877*
rs754615	C	0.3907	-0.05297	0.09947	-0.53	*0.5944*
rs1862609	A	0.3129	-0.09579	0.10434	-0.92	*0.3586*
rs27772	G	0.3133	-0.10841	0.10463	-1.04	*0.3001*
rs28081	A	0.1510	0.08516	0.13473	0.63	*0.5273*
rs27037	A	0.2957	0.19972	0.10564	1.89	*0.0587*
rs27429	C	0.0598	0.32834	0.20469	1.60	*0.1087*
rs27524	A	0.3730	0.19677	0.10002	1.97	*0.0492*
rs25862	A	0.4488	0.00072	0.09761	0.01	*0.9941*
rs11135480	C	0.1321	0.16456	0.14237	1.16	*0.2478*
rs10515247	A	0.1323	0.15257	0.14235	1.07	*0.2838*
rs149078	A	0.2965	-0.03113	0.10559	-0.29	*0.7681*
rs27042	A	0.3655	0.06812	0.10044	0.68	*0.4977*
rs27044	G	0.2795	0.05808	0.10775	0.54	*0.5899*
rs17482078	A	0.2039	-0.12010	0.11979	-1.00	*0.3161*
rs42398	G	0.1470	0.01970	0.13619	0.14	*0.8850*
rs469783	G	0.4374	0.12081	0.09810	1.23	*0.2181*
rs10050860	A	0.2127	-0.11031	0.11847	-0.93	*0.3518*
rs13154629	A	0.2131	-0.12626	0.11771	-1.07	*0.2834*
rs30187	A	0.3478	0.11181	0.10165	1.10	*0.2714*
rs27434	A	0.2175	0.05464	0.11888	0.46	*0.6458*
rs26618	G	0.2370	0.01643	0.11399	0.14	*0.8854*
rs25866	A	0.2341	0.05658	0.11692	0.48	*0.6285*
rs26653	C	0.2835	0.10223	0.10743	0.95	*0.3413*
rs34753	G	0.2796	0.09403	0.10795	0.87	*0.3837*
rs28129	G	0.2804	0.10265	0.10786	0.95	*0.3413*
rs18036	G	0.2441	0.09210	0.11311	0.81	*0.4155*
rs152280	A	0.2085	0.03860	0.11923	0.32	*0.7461*
rs12520537	G	0.1537	0.10012	0.13468	0.74	*0.4573*
rs41135	G	0.4669	-0.06933	0.09719	-0.71	*0.4756*
rs34736	A	0.0648	0.22406	0.19710	1.14	*0.2557*
ERAP2						
rs2911132	A	0.3726	-0.06134	0.10034	-0.61	*0.5410*
rs2042381	A	0.2733	-0.01152	0.10842	-0.11	*0.9154*
rs2927615	A	0.2404	-0.16903	0.11295	-1.50	*0.1346*
rs2927612	G	0.1168	0.21419	0.15033	1.42	*0.1542*
rs2549778	A	0.4292	0.08892	0.09765	0.91	*0.3626*
rs6861666	G	0.0781	0.13711	0.18020	0.76	*0.4467*
rs3733904	G	0.2117	-0.31140	0.11921	-2.61	*0.0090*
rs2549779	G	0.4880	-0.13241	0.09735	-1.36	*0.1738*
rs4869315	A	0.4241	-0.24981	0.09804	-2.55	*0.0108*
rs2549782	C	0.4093	0.20951	0.09767	2.15	*0.0320*
rs17408150	T	0.0560	0.08593	0.21187	0.41	*0.6851*
rs7714122	G	0.0648	0.07624	0.19807	0.38	*0.7003*

Adjusted for age, body-mass index, history of diabetes, history of hyperlipidemia, current smoking, exercise, alcohol use, education level, current hormone use.

SE=standard error.

Online Supplementary Data Table 2: Linear regression analysis for all SNPs evaluated with baseline diastolic blood pressure levels

dbSNP	MA	MAF	Beta-estimate	SE	t-Value	*p*-uncorrected
ERAP1						
rs1559085	G	0.1413	-0.05589	0.11584	-0.48	*0.6295*
rs27851	G	0.0607	0.44767	0.16965	2.64	*0.0083*
rs3756623	C	0.0750	0.02906	0.15346	0.19	*0.8498*
rs754615	C	0.3907	-0.00069	0.08300	-0.01	*0.9934*
rs1862609	A	0.3129	-0.01928	0.08706	-0.22	*0.8248*
rs27772	G	0.3133	-0.06726	0.08732	-0.77	*0.4411*
rs28081	A	0.1510	0.07781	0.11238	0.69	*0.4887*
rs27037	A	0.2957	0.08035	0.08806	0.91	*0.3616*
rs27429	C	0.0598	0.44405	0.17080	2.60	*0.0093*
rs27524	A	0.3730	0.08188	0.08349	0.98	*0.3267*
rs25862	A	0.4488	0.09248	0.08143	1.14	*0.2561*
rs11135480	C	0.1321	-0.02839	0.11882	-0.24	*0.8112*
rs10515247	A	0.1323	-0.03785	0.11877	-0.32	*0.7500*
rs149078	A	0.2965	-0.09694	0.08815	-1.10	*0.2715*
rs27042	A	0.3655	0.09181	0.08386	1.09	*0.2736*
rs27044	G	0.2795	-0.02894	0.08990	-0.32	*0.7475*
rs17482078	A	0.2039	-0.03560	0.09996	-0.36	*0.7217*
rs42398	G	0.1470	0.02119	0.11360	0.19	*0.8520*
rs469783	G	0.4374	0.00445	0.08184	0.05	*0.9566*
rs10050860	A	0.2127	-0.04753	0.09887	-0.48	*0.6307*
rs13154629	A	0.2131	-0.04100	0.09823	-0.42	*0.6764*
rs30187	A	0.3478	0.02449	0.08479	0.29	*0.7727*
rs27434	A	0.2175	0.06346	0.09912	0.64	*0.5220*
rs26618	G	0.2370	-0.07185	0.09509	-0.76	*0.4499*
rs25866	A	0.2341	0.08557	0.09757	0.88	*0.3805*
rs26653	C	0.2835	0.14834	0.08963	1.66	*0.0979*
rs34753	G	0.2796	0.14765	0.09012	1.64	*0.1014*
rs28129	G	0.2804	0.15353	0.08999	1.71	*0.0880*
rs18036	G	0.2441	-0.02800	0.09437	-0.30	*0.7667*
rs152280	A	0.2085	0.04775	0.09951	0.48	*0.6314*
rs12520537	G	0.1537	0.08176	0.11238	0.73	*0.4670*
rs41135	G	0.4669	-0.09209	0.08110	-1.14	*0.2562*
rs34736	A	0.0648	0.39074	0.16446	2.38	*0.0175*
ERAP2						
rs2911132	A	0.3726	0.01459	0.08372	0.17	*0.8617*
rs2042381	A	0.2733	-0.06922	0.09042	-0.77	*0.4440*
rs2927615	A	0.2404	-0.08548	0.09427	-0.91	*0.3646*
rs2927612	G	0.1168	0.03659	0.12543	0.29	*0.7705*
rs2549778	A	0.4292	0.05886	0.08151	0.72	*0.4703*
rs6861666	G	0.0781	0.07794	0.15049	0.52	*0.6045*
rs3733904	G	0.2117	-0.08272	0.09947	-0.83	*0.4056*
rs2549779	G	0.4880	-0.07126	0.08121	-0.88	*0.3802*
rs4869315	A	0.4241	-0.08793	0.08181	-1.07	*0.2825*
rs2549782	C	0.4093	0.08601	0.08154	1.05	*0.2915*
rs17408150	T	0.0560	-0.17605	0.17683	-1.00	*0.3195*
rs7714122	G	0.0648	-0.17078	0.16523	-1.03	*0.3014*

Adjusted for age, body-mass index, history of diabetes, history of hyperlipidemia, current smoking, exercise, alcohol use, education level, current hormone use.
SE=standard error.

Online Supplementary Data Table 3: Expression quantitative trait loci (eQTL) of SNPs associated with blood pressure progression and incident hypertension -- eQTL from mRNA expression of otherwise normal human cardiovascular tissues based on the *GTEx Analysis Release V6p (dbGaP accession phs000424.v6.p1)

Gene	Gencode ID	SNP	*p*	ES	T	SE	Tissue
ERAP1	ENSG00000164307.8	rs27524	0.0042	-0.35	-2.9	0.12	Artery - Coronary
		rs27851	0.043	0.39	2	0.19	Artery - Coronary
		rs27429	0.08	-0.36	-1.8	0.2	Artery - Coronary
		rs30187	0.00022	-0.44	-3.8	0.11	Artery - Coronary
		rs34736	0.1	-0.32	-1.7	0.19	Artery - Coronary
		rs27772	0.052	-0.24	-2	0.12	Artery - Coronary
		rs469783	0.0042	-0.33	-2.9	0.11	Artery - Coronary
		rs10050860	0.88	-0.025	-0.16	0.16	Artery - Coronary
		rs27524	0.00087	-0.21	-3.4	0.062	Artery - Tibial
		rs27851	0.0023	0.37	3.1	0.12	Artery - Tibial
		rs27429	0.0025	-0.4	-3.1	0.13	Artery - Tibial
		rs30187	0.000073	-0.24	-4	0.06	Artery - Tibial
		rs34736	0.00016	-0.46	-3.8	0.12	Artery - Tibial
		rs27772	1.40E-13	-0.44	-7.8	0.056	Artery - Tibial
		rs469783	1.30E-07	-0.3	-5.4	0.056	Artery - Tibial
		rs10050860	0.0000086	0.35	4.5	0.076	Artery - Tibial
		rs27524	0.000015	-0.4	-4.5	0.088	Adrenal Gland
		rs27851	0.13	0.25	1.5	0.17	Adrenal Gland
		rs27429	0.53	-0.12	-0.64	0.19	Adrenal Gland
		rs30187	0.00001	-0.44	-4.6	0.094	Adrenal Gland
		rs34736	0.23	-0.22	-1.2	0.19	Adrenal Gland
		rs27772	0.00028	-0.33	-3.8	0.088	Adrenal Gland
		rs469783	0.002	-0.3	-3.2	0.096	Adrenal Gland
		rs10050860	0.18	0.18	1.3	0.13	Adrenal Gland
		rs27524	2.90E-07	-0.36	-5.4	0.066	Heart - Left Ventricle
		rs27851	0.66	-0.06	-0.44	0.14	Heart - Left Ventricle
		rs27429	0.55	0.088	0.59	0.15	Heart - Left Ventricle
		rs30187	2.20E-07	-0.36	-5.4	0.066	Heart - Left Ventricle
		rs34736	0.58	0.085	0.55	0.15	Heart - Left

							Ventricle
		rs27772	0.18	-0.1	-1.4	0.075	Heart - Left Ventricle
		rs469783	0.00075	-0.24	-3.4	0.069	Heart - Left Ventricle
ERAP2	ENSG00000164308.12	rs3733904	0.000042	-0.63	-4.3	0.15	Artery - Coronary
		rs4869315	1.40E-15	-0.85	-9.6	0.089	Artery - Coronary
		rs2549782	2.10E-25	-1.1	-14	0.077	Artery - Coronary
		rs2927615	0.39	-0.15	-0.86	0.17	Artery - Coronary
		rs3733904	2.00E-08	-0.62	-5.8	0.11	Artery - Tibial
		rs4869315	1.60E-32	-0.84	-14	0.061	Artery - Tibial
		rs2549782	1.10E-72	-1.1	-26	0.042	Artery - Tibial
		rs2927615	0.64	-0.045	-0.46	0.097	Artery - Tibial
		rs3733904	0.0029	-0.53	-3	0.17	Adrenal Gland
		rs4869315	1.90E-12	-0.9	-8	0.11	Adrenal Gland
		rs2549782	5.80E-24	-1.1	-13	0.08	Adrenal Gland
		rs2927615	0.75	0.06	0.31	0.19	Adrenal Gland
		rs3733904	0.000021	-0.49	-4.4	0.11	Heart - Left Ventricle
		rs4869315	6.60E-20	-0.75	-11	0.071	Heart - Left Ventricle
		rs2549782	1.50E-44	-0.99	-20	0.049	Heart - Left Ventricle
		rs2927615	0.51	-0.085	-0.66	0.13	Heart - Left Ventricle

SNP=single nucleotide polymorphism, ES=effect size, T=T-statistic, SE=standard error
*GTEx Portal (http://www.gtexportal.org/home/testyourown)

Online Supplementary Data Table 4: Genotype-phenotype association of ERAP 1 and 2 gene variants with blood pressure as described in dbGaP

dbSNP	MA**	IβI	*p*-value
*rs34753	C	0.0223	0.7123
*rs28129	G	0.0223	0.7123
*rs18036	C	0.0944	0.1530
rs151823	A	0.0656	0.5103
*rs152280	T	0.1158	0.0795
rs34733	T	0.4041	0.0003
*rs12520537	G	0.1415	0.0766
*rs41135	C	0.0456	0.4376
rs152468	T	0.0874	0.2677
*rs34736	T	0.4045	0.0003
rs11748795	T	0.0722	0.3993
rs10476692	C	0.0337	0.7802
rs845028	G	0.0823	0.2291
rs7708451	T	0.4595	0.000089
*rs2911132	A	0.0296	0.6065
rs11750671	C	0.0774	0.3701
*rs2042381	A	0.1055	0.0960
*rs2927615	A	0.1117	0.0873
rs2927613	A	0.1323	0.0239
*rs2927612	G	0.1335	0.1300
rs2911134	G	0.0667	0.2497
rs2549778	T	0.0636	0.2802
rs4092590	C	0.1182	0.1049
rs6861666	G	0.2711	0.0092

dbGaP=The database of Genotypes and Phenotypes (www.ncbi.nlm.nih.gov/gap)
IβI=absolute beta-estimate
*dbSNPs were evaluated in the present study
**MA=minor allele as defined by the Phenotype-Genotype Integrator in dbGaP
All values presented are direct extracts from the Phenotype-Genotype Integrator in dbGaP

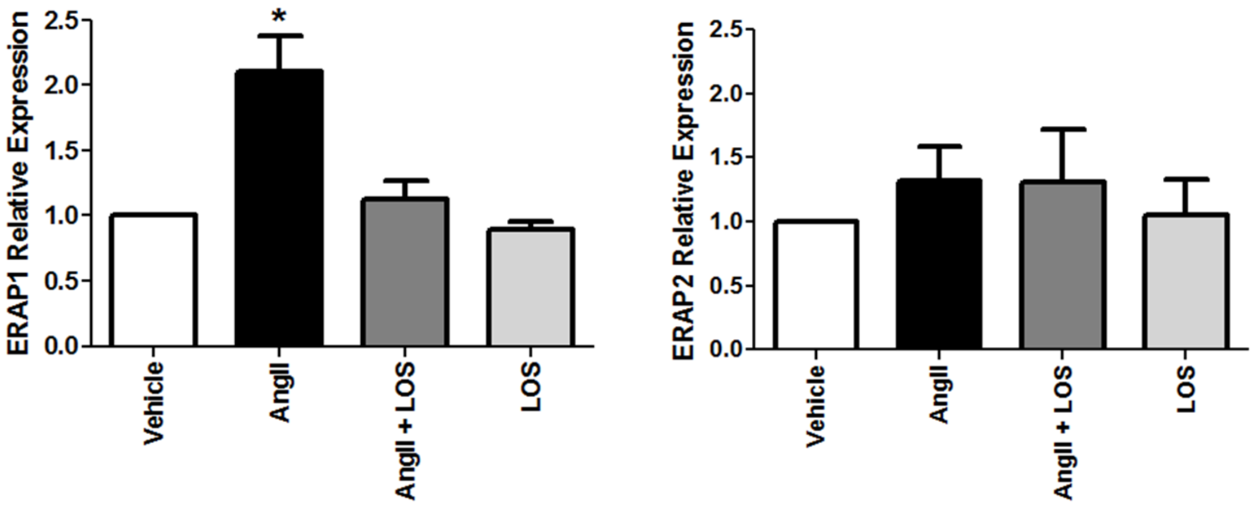

Online Supplementary Fig 1. Stimulation of EA.hy926 endothelial cells with AngII [10nM, 24h] leads to increases in ERAP1 but not ERAP2 gene expression. Increases in ERAP1 gene expression were blocked by Losartan [LOS, 1μM], an AngII type 1 receptor antagonist. Data show mean ± SE. (*P<0.05 vs. Vehicle or AngII + LOS, n=4 in triplicate determinations, ANOVA, Tukey's multiple comparison tests).
